# Optimising individualised treatment decisions for locally advanced thymoma: precision selection of minimally invasive surgery and postoperative adjuvant radiotherapy

**DOI:** 10.3389/fonc.2026.1839919

**Published:** 2026-06-11

**Authors:** Xuyang Peng, Qi Zhang, Bin Huang, Jianyang Ding, Mingjiang Huang, Chaofan Mao, Xuhui Wu, Xi Zhu

**Affiliations:** 1 Department of Cardiothoracic Surgery, The First Affiliated Hospital of Lishui University, Lishui People's Hospital, Lishui, Zhejiang, China; 2 Hangzhou Normal University, Hangzhou, Zhejiang, China; 3 Department of Head and Neck Surgery, the Fifth Hospital Affiliated to Wenzhou Medical University, Lishui Central Hospital, Lishui, Zhejiang, China

**Keywords:** individualised treatment, locally advanced thymoma, minimally invasive surgery, multidisciplinary team, postoperative radiotherapy

## Abstract

Locally advanced thymoma is characterised by significant heterogeneity and complex biological behaviour, and its optimal treatment strategy has long been controversial. The integration of minimally invasive surgical techniques and advanced radiotherapy technologies is refining the standard treatment mode towards individualised decision-making. This article reviews the latest evidence-based medical progress regarding the selection of surgical approaches and postoperative adjuvant treatment decisions for locally advanced thymoma in recent years. Regarding surgical treatment, the application boundaries of robotic and thoracoscopic minimally invasive surgery in locally advanced cases are analysed, indicating that when complete tumour resection (R0) is ensured, minimally invasive surgery can yield oncological outcomes non-inferior to those of open surgery and significantly reduce perioperative complications. Regarding radiation therapy, the evolution of indications for postoperative radiotherapy (PORT) is discussed, emphasising the critical role of risk stratification models based on WHO histological classification, resection margin status, and tumour burden in guiding radiotherapy decisions, and the potential of new technologies such as proton therapy in reducing cardiac toxicity is evaluated. This article aims to construct a dynamic individualised treatment decision-making framework by integrating multidisciplinary perspectives, providing a theoretical basis and practical guidance for clinicians to balance oncological efficacy and patient quality of life.

## 1 Introduction

Thymic epithelial tumours (TETs) are rare but represent the most common malignancies in the anterior mediastinum, necessitating precise clinical management (1). According to the World Health Organization (WHO) classification, thymic epithelial tumours mainly include thymomas (divided into types A, AB, B1, B2, and B3), thymic carcinomas, and rare thymic neuroendocrine tumours (2, 3). Although the annual incidence of these tumours is relatively low, they can occur at any age, with the average age at diagnosis being 40–50 years (4). Based on the U.S. SEER database and German cancer registry data, the age-standardised incidence rates of thymoma in the United States and Germany are 2.2/million and 2.64/million, respectively, while those for thymic carcinoma are 0.48/million and 0.42/million, respectively (5); furthermore, data from the Korea Central Cancer Registry show that the average annual percentage change (AAPC) in the overall incidence of TETs reached 6.1%, with AAPCs for thymoma and thymic carcinoma being 5.6% and 7.0%, respectively, suggesting that the incidence in the Asian population is significantly higher than in Europe and America (6). Regarding gender distribution, the male-to-female ratio for thymoma is close to 1:1 (U.S. data: 1:1.09), whereas the incidence of thymic carcinoma is higher in females (U.S. male-to-female ratio: 1:1.61) (5). The 5-year net survival (NS) rate for thymoma reaches 82.3%, while it is only 46.2% for thymic carcinoma (6), emphasising the necessity for precision management of different subtypes (7).

Advanced thymic epithelial tumours are explicitly defined within the Masaoka-Koga staging system as locally invasive tumours, specifically including stage IIb lesions characterised by macroscopic invasion into the thymic capsule or surrounding fatty tissue; stage III tumours accompanied by macroscopic invasion into neighbouring organs (such as the pericardium, great vessels, or lung); and stage IV diseases characterised by pleural or pericardial dissemination, lymph node involvement, or distant metastasis (8, 9). Such locally advanced or metastatic tumours often manifest as infiltration of surrounding structures (e.g., pericardium, great vessels, lung parenchyma, etc.), regional lymph node invasion, or systemic spread. Due to their strong invasiveness and complex treatment strategies, a multimodal comprehensive regimen comprising surgery, radiotherapy, chemotherapy, and immunotherapy is required, which is directly correlated with patients’ long-term survival outcomes. Consequently, accurately identifying the stage and invasive characteristics of advanced thymic tumours is fundamental for optimising individualised treatment strategies and improving prognosis.

Traditionally, the treatment of locally advanced thymoma has centred on open surgery (such as median sternotomy), aiming to achieve complete resection (R0), and is often supplemented with postoperative radiotherapy or chemotherapy to reduce the risk of recurrence (4, 10). However, this paradigm is subject to significant controversy: the application of adjuvant radiotherapy lacks uniform standards, especially for completely resected early-stage tumours (e.g., Masaoka-Koga stage II), where evidence suggests uncertain survival benefits, potentially leading to overtreatment or undertreatment in some patients. For instance, retrospective studies indicate that postoperative radiotherapy has limited local control effects on thymoma, whereas thymic carcinoma might benefit more, yet this “all-or-nothing” strategy ignores tumour biological heterogeneity (4). These controversies highlight the limitations of traditional therapies regarding insufficient individual risk stratification.

In recent years, the maturation of minimally invasive surgery (MIS) techniques (such as thoracoscopic or robotic-assisted methods) and the development of precise prognostic indicators have jointly driven the transformation of treatment modes towards individualization. While MIS is increasingly recognised as the preferred standard approach for early-stage thymomas—and even for tumours larger than 5 cm in experienced centres—current international guidelines still conservatively recommend open sternotomy as the standard of care for locally advanced cases (Masaoka-Koga stage III/IVa). Therefore, this review aims to challenge this traditional boundary, highlighting that MIS can also be safely applied to selected locally advanced thymomas without compromising the principle of complete resection. Meanwhile, molecular features (e.g., *KIT* mutations, PD-L1 expression, *GTF2I* mutations) and advances in imaging (e.g., high uptake of FDG-PET in thymic carcinoma) provide new tools for risk stratification (2, 11), allowing treatment decisions to be based on tumour biological behaviour rather than purely on staging. Korean registry data also show an increase in the proportion of early detection of thymoma, partly attributed to improved screening practices, further supporting the feasibility of individualised management (6). This transformation aims to balance efficacy and toxicity to optimise patient quality of life.

This review systematically integrates evidence-based data on MIS and postoperative radiotherapy for the first time to construct a decision-making framework based on multidimensional risk stratification (9). By reviewing clinical guidelines and clinical studies, we combine surgical feasibility, radiotherapy indications, and molecular markers to provide a theoretical basis for the individualised pathway of locally advanced thymoma, which is expected to bridge the gaps in the traditional paradigm. This framework aims to guide clinical decision-making and inform future research directions.

## 2 Evolution of the role of minimally invasive thymectomy in locally advanced thymoma: from contraindication to feasible option

### 2.1 Technical overview and comparison

With the continuous evolution of MIS techniques, the surgical treatment of thymoma has expanded from traditional open surgery to various minimally invasive methods, including robotic-assisted thoracoscopic surgery (RATS), video-assisted thoracoscopic surgery (VATS), and the subxiphoid approach. These techniques aim to optimise patient prognosis by reducing surgical trauma; this section will outline their technical characteristics and theoretically compare them with open surgery to provide a basis for subsequent efficacy evaluation (12).

Minimally invasive thymectomy is defined as any surgical method that does not involve sternotomy or rib spreading, and its core lies in achieving complete resection of the tumour through video monitoring (13). RATS utilises the da Vinci robotic system to provide a three-dimensional magnified view and instrument flexibility with seven degrees of freedom, effectively filtering hand tremors and facilitating precise operation within the narrow mediastinal space, making it particularly suitable for thymoma resection (14, 15). Compared with VATS, the learning curve for RATS is shorter, requiring approximately 15–20 cases to master, but equipment costs and setup times are longer (14). VATS achieves two-dimensional visualisation through a thoracoscope and typically employs three-port or uniportal techniques; although operational flexibility is lower, it remains a common choice for early-stage thymoma, especially for cases with smaller tumour diameters (14, 16). Additionally, the subxiphoid approach, as an emerging technique, accesses the mediastinum through a subxiphoid incision, providing a view similar to sternotomy that facilitates bilateral exploration and upper mediastinal exposure, but the working space is limited and the learning curve is steeper (17, 18). These techniques each have advantages; the high precision of RATS and the popularity of VATS make them effective alternatives to open surgery, but the subxiphoid approach still requires more long-term data to verify its stability (12).

Theoretically, MIS and open surgery differ in several key aspects. MIS techniques usually involve less trauma, shorter postoperative hospital stays, and significantly reduced intraoperative blood loss (19, 20). For example, both RATS and VATS can achieve complete tumour resection rates comparable to open surgery, but MIS methods have advantages in reducing intraoperative blood loss, shortening hospital stay, and lowering postoperative complications (such as surgical site infection and pneumonia) (15, 16, 21). Although open surgery provides better exposure and is suitable for advanced tumours, it involves greater trauma and longer hospital stays, and sternotomy-related complications such as infection and healing issues are higher (14). However, open surgery still holds advantages in managing large or invasive tumours, especially in cases requiring vascular reconstruction or extensive lymph node dissection (12). Regarding the learning curve, RATS and VATS require a certain accumulation of cases, while open surgery techniques are mature, yet the overall recovery advantages of MIS are significant (18, 22). These findings collectively indicate that MIS techniques may be superior to open surgery in selected cases, but individualised decisions need to be made based on tumour size, extent of invasion, and surgical experience.

### 2.2 Updated evidence on oncological outcomes

In recent years, with the maturation of technology and the accumulation of experience, the application of MIS in the treatment of locally advanced thymoma has evolved from exploratory attempts to a viable option for strictly selected cases. Substantial evidence indicates that, provided patients are meticulously selected by experienced centres (e.g., based on tumour size, relationship with great vessels, and specific modes of invasion), MIS can achieve oncological outcomes comparable to traditional open surgery. This section will integrate recent key research evidence (detailed data are shown in **Table 1**), focussing on analysing the similarities and differences in findings across different study designs, thereby forming a more comprehensive understanding of the oncological non-inferiority of MIS.

**Table 1:** Summary of oncological outcomes of minimally invasive surgery and open surgery for thymoma.

Author (Year)	Study type	Subjects	Follow-up time	R0 resection rate (%)	Recurrence-Free survival (RFS)	Overall survival (OS)	Other outcomes	Ref
Gu et al	Single-centre Retrospective Analysis	AJCC/UICC/IASLC/ITMIG stage T2-3NxM0 thymic malignancies, n=128 (MIS group: 50, Open Thymectomy group: 70; 40 each after PSM)	Median follow-up: MIS group 46.5 months, Open Thymectomy group 88 months	MIS: 100%, Open Thymectomy: 100%	5-year FFR (similar to RFS): MIS 78.2%, Open Thymectomy 78.5% (p=0.942)	MIS: 100%, Open Thymectomy: 100%	Multivariable analysis: Surgical approach was not an independent predictor of recurrence (HR = 0.922, 95%CI 0.332-2.559, p=0.727)	(23)
Sicolo et al	Multicentre Retrospective Study	Thymoma patients with myasthenia gravis, n=213	Overall mean follow-up 58 months (SD ± 36), Open Thymectomy group 78 months (SD ± 32), RATS group 39 months (SD ± 28)	RATS: 98%, Open Thymectomy: 97.3% (p=0.705)	5-year DFS: Entire cohort 96%, between-group p=0.937	5-year OS: RATS 87%, Open Thymectomy 86% (p=0.701)	–	(24)
Yang et al	Large-scale NCDB-based Study	Stage I-III thymoma patients, n=1223 (MIS: 317, Open Thymectomy: 906; 185 pairs after PSM)	Median follow-up: Open Thymectomy group 40.7 months (IQR, 27.3-56.8), MIS group 35.9 months (IQR, 24.9-52.2)	MIS: 72.2%, Open Thymectomy: 67.7% (p=0.62)	–	5-year OS: MIS 89.4%, Open Thymectomy 81.6% (p=0.20)	–	(25)
Comacchio et al	Multicentre Nationwide Study	Thymic epithelial tumour patients, n=669 (underwent robotic thymectomy)	Median follow-up 29 months	98.60%	5-year recurrence rate 7.4%, 10-year recurrence rate 8.3%	5-year OS 94%, 10-year OS 77%	–	(26)
Patel et al	Multicentre Study (PSM analysis)	Thymoma patients, n=732	Median follow-up: VATS group 50 months, RATS group 40 months	RATS: 98.3%, VATS: 94.7% (OR = 0.227, 95%CI 0.09-0.551, P<0.001)	RFS: HR = 1.27, P = 0.553	OS: HR = 0.95, P = 0.908	–	(27)
Odaka et al	Retrospective Single-centre Study	Masaoka stage I-IVa thymoma patients, n=140 (TT group: 88, OT group: 52)	Overall median follow-up 59 months (TT group: 54 months, OT group: 99 months)	100%	5-year DFS: TT 88.0% (95%CI 77-93%), OT 84.2% (95%CI 70-92%) (P = 0.3906)	–	Multivariable analysis: Masaoka stage III-IV (HR = 12.36, 95%CI 1.58-269.80, P = 0.0141) and WHO type B3-C (HR = 7.17, 95%CI 1.02-155.30, P = 0.0471) were independent prognostic factors for DFS	(28)
Odaka et al	Study focussing on large thymomas	Thymoma patients, n=135 (tumour ≥50mm group: 63, TT: 38, OT: 25)	Median postoperative follow-up 66.2 months (range 5–210 months)	100%	5-year DFS: TT 92.4%, OT 86.67% (P = 0.3501)	–	Multivariable analysis: Masaoka stage III-IV was an independent risk factor (HR = 16.38, 95%CI 3.82-98.98, P<0.0001); tumour size did not significantly affect prognosis	(29)
Chen et al	Small-sample Retrospective Study	Masaoka stage III thymic epithelial tumour patients, n=26 (VATS group: 8, Open Thymectomy group: 18)	Mean follow-up: VATS group 8 months, Open Thymectomy group 15 months	100%	No recurrence during short-term follow-up	–	tumour size >6cm, invasion of phrenic nerve or superior vena cava significantly hindered successful VATS (P<0.05)	(30)
Burt et al	Multicentre Analysis based on ITMIG database	Thymic malignancy patients, n=2514 (after PSM: MIS group 266, Open Thymectomy group 266)	–	MIS: 96%, Open Thymectomy: 96% (P = 0.7)	–	–	Multivariable analysis: Surgical approach was not an independent predictor of R0 resection (OR = 1.32, P = 0.42)	(31)
Zhang et al	Single-centre Cohort Study	Stage II-IV thymoma patients, n=41 (Robotic group: 20, Open Thymectomy group: 21)	Median follow-up 34.4 months	–	Recurrence rate: Robotic 15% (3/20), Open Thymectomy 38.1% (P = 0.10)	No mortality events	–	(32)
Davis et al	Retrospective Cohort Study	Stage I-III thymoma patients, n=178 (OT group: n=129, 72%; MIS group: n=49, 28%)	Median follow-up 64 months (IQR, 26.3-112.8)	Overall: 82% (OT: 83%, MIS: 80%; p=0.76)	5-year DFS: OT 67.6%, MIS 58.5%; 10-year DFS: OT 54.3%, MIS 51.2% (unweighted analysis, p=0.52). Multivariable analysis: MIS associated with worse DFS (HR = 2.481, 95% CI 1.31-4.71, p=0.005)	5-year OS: OT 91.8%, MIS 95.8%; 10-year OS: OT 83.2%, MIS 85.6% (unweighted analysis, p=0.68). Multivariable analysis: No significant difference in OS (HR = 0.742, p=0.56)	–	(33)
Hurd et al	National Cancer Database-based Study	Stage I-III thymic carcinoma patients, n=216 (after PSM)	Median follow-up: Open Thymectomy group 57.2 months (IQR 36.0-78.4), MIS group 59.0 months (IQR 36.2-83.6)	Negative margin rate: Open Thymectomy 54.9% (95/173), MIS 60.5% (26/43)	–	5-year OS: MIS 78.6%, Open Thymectomy 54.6% (p=0.15)	–	(34)

AJCC, American Joint Committee on Cancer; UICC, Union for International Cancer Control; IASLC, International Association for the Study of Lung Cancer; ITMIG, International Thymic Malignancy Interest Group; MIS, Minimally Invasive Surgery; PSM, Propensity Score Matching; RATS, Robot-Assisted Thoracic Surgery; VATS, Video-Assisted Thoracic Surgery; TT, Total Thoracoscopic (surgery); OT, Open Thoracotomy; R0 resection, Complete resection with microscopically negative margins; FFR, Freedom From Recurrence; DFS, Disease-Free Survival; RFS, Recurrence-Free Survival; OS, Overall Survival; HR, Hazard Ratio; CI, Confidence Interval; OR, Odds Ratio; IRR, Incidence Rate Ratio; IQR, Interquartile Range; SD, Standard Deviation; NCDB, National Cancer Database (US); ESTS, European Society of Thoracic Surgeons.

Overall, the current body of evidence strongly supports the oncological safety of MIS in selected patients with locally advanced thymoma. Although sample sizes were limited, multiple single-centre studies have controlled for confounding factors through methods such as propensity score matching, providing robust preliminary evidence. These studies (23, 28–30, 32) consistently indicate that for patients with Masaoka stage III or even selected stage IVa disease, as well as those with larger tumour sizes, thoracoscopic or robotic-assisted thymectomy can achieve R0 resection rates, recurrence-free survival (RFS), and overall survival (OS) similar to those of open surgery. These single-centre studies laid the foundation for the non-inferiority of MIS and emphasised the pivotal role of precise patient selection.

Multicentre studies and large-scale database analyses have further strengthened the generalisability of the above conclusions. By including more cases and data from multiple centres, these studies (24–27, 31) effectively reduced the potential bias inherent in single-centre studies. The results similarly showed that after matching for baseline characteristics, there were no statistically significant differences between the MIS group and the open surgery group in terms of R0 resection rate, recurrence rate, and survival rate. Notably, one study (27) found that the R0 resection rate of robotic surgery was superior to that of thoracoscopic surgery, yet the two remained equivalent in terms of long-term survival outcomes, suggesting that technological advancements may lead to improvements in surgical quality. These large-scale real-world studies collectively indicate that the oncological equivalence of MIS is not a technical exception confined to individual centres but a result reproducible on a broader scale.

Large-scale studies based on the National Cancer Database (NCDB) have provided important real-world evidence for evaluating the oncological outcomes of MIS. For example, an analysis by Hurd et al. (34) focussing on patients with thymic carcinoma showed no statistically significant difference in long-term survival rates between MIS and open surgery. However, existing evidence remains heterogeneous and occasionally contradictory. An important piece of controversial evidence comes from the study by Davis et al. (33), in which multivariate analysis results showed that MIS was associated with poorer DFS, despite no difference in overall survival. This discrepancy suggests that results may vary across different patient populations, surgical techniques, or follow-up durations, and also reflects the inherent limitations of retrospective studies.

In summary, the majority of existing evidence points to a consistent conclusion: in strictly selected patients with locally advanced thymoma, MIS can achieve oncological outcomes comparable to open surgery. The key to its success lies in case selection rather than the surgical approach itself. The main discrepancy is reflected in the concerns raised by a few studies regarding disease-free survival, which may be attributable to residual confounding bias or differences in patient risk stratification. The limitations of current evidence—including the fact that most studies are retrospective, the potential for selection bias, and the fact that follow-up times in some studies are still insufficient to capture the very late recurrence characteristics of thymoma—require caution during interpretation. Therefore, clinical decision-making should be based on multidisciplinary discussions, integrating tumour stage, pathological type, extent of invasion, and the experience of the surgical team to individualise the selection of the surgical approach. Future prospective studies are expected to provide more definitive evidence for the application of MIS in higher-risk patients.

### 2.3 Patient selection and surgical contraindications

For patients with locally advanced (Masaoka-Koga stage III and selected stage IVa) thymoma, the application of MIS is no longer an absolute contraindication; however, clinical decision-making must be established on a precise assessment of the anatomical extent of tumour invasion and biological behaviour. Existing evidence supports that in strictly selected cases, MIS, as a feasible treatment modality, can achieve oncological prognoses comparable to open surgery, but this is highly dependent on a strict adherence to surgical boundaries (23). Therefore, refining surgical indications from traditional “anatomical resectability” to a stratified selection based on invaded structures and technical feasibility is a core component of optimising individualised treatment strategies.

Regarding the characteristics of ideal candidates for MIS, existing evidence emphasises the importance of “limited invasion.” Specifically, patients with tumours showing only limited invasion of the pericardium, lung tissue, or unilateral phrenic nerve are considered suitable candidates for MIS. For instance, the study by Gu et al., through the construction of a Resection Index, indicated that combined resection involving the pericardium (score 1) or lung and phrenic nerve (score 2) can usually be safely completed under thoracoscopy without increasing perioperative risk (23). Furthermore, regarding screening criteria for tumour size, although controversy exists, the study by Chen et al. explicitly pointed out that tumours smaller than 6 cm in diameter are more suitable for the minimally invasive approach; for strictly selected Masaoka stage III thymomas, even if the tumour invades the innominate vein, it is not an absolute contraindication for MIS, and R0 resection can still be attempted and completed under thoracoscopy (30). Dhamija et al. further supplemented that even with left innominate vein involvement, minimally invasive resection can be safely attempted in experienced centres. Traditional contraindications (such as extensive adhesion to surrounding structures) are not absolute but require comprehensive judgement through preoperative assessment and technical experience; complete resection of the left innominate vein is feasible under specific conditions (e.g., no history of neck surgery) (35). These findings collectively indicate that for locally advanced cases with limited invasion scope and clear anatomical relationships, MIS should be recommended as the first choice.

However, clinical practice frequently encounters “grey zone” scenarios that complicate preoperative assessment and the surgeon’s technical learning curve. Regarding the boundary of tumour size, although Chen et al. (30) suggested a cutoff of 6 cm, subsequent studies focussing on large tumours pointed out that for tumours with a diameter greater than 5 cm or even larger, provided there is no extensive invasion of deep mediastinal structures, MIS is technically feasible and does not significantly increase the risk of recurrence (28, 36). This suggests that tumour size itself is not an absolute limitation; the key lies in its anatomical relationship with surrounding critical structures. Additionally, the decision regarding preoperative biopsy is also one of the challenges faced at this stage. A large-sample retrospective analysis by Davis et al. found that preoperative image-guided needle biopsy or thoracoscopic biopsy was independently associated with poorer DFS, which may be related to the potential risk of dissemination caused by disruption of the tumour capsule; therefore, unnecessary preoperative invasive procedures should be avoided for resectable lesions with typical imaging characteristics (33). Meanwhile, team experience is also a key factor determining the ability to navigate the “grey zone.” Research by Zhang et al. indicated that with the accumulation of surgical cases (e.g., exceeding 30 cases of robotic surgery experience), the rate of conversion to open surgery significantly decreased, and operative time shortened. This emphasises that high-difficulty surgeries for locally advanced thymoma should be performed by experienced surgeons to ensure technical safety and efficacy (32).

Despite continuous advancements in MIS techniques, based on principles of oncological safety, specific anatomical invasion characteristics should still be considered absolute contraindications (surgical “no-go” zones) for MIS. Zhang et al., in their study on locally advanced thymic malignancies, constructed a clear decision tree, pointing out that when imaging shows tumour encasement of the aorta or pulmonary artery exceeding 50%, or encasement of the superior vena cava and left brachiocephalic vein exceeding 30% with disappearance of the fat layer, open surgery should be the preferred choice (32). Similarly, data from Chen et al. confirmed that simultaneous involvement of the superior vena cava and phrenic nerve is a significant risk factor precluding the successful completion of thoracoscopic surgery (30). Furthermore, extensive invasion of deep mediastinal structures such as the trachea, oesophagus, or myocardium is also regarded as a contraindication for robotic surgery (36). Additionally, for deep invasion of hilar structures, due to the high difficulty of vascular control during MIS, it is listed as a direct indication for open surgery; if discovered unexpectedly intraoperatively, a planned conversion to open surgery is required (32).

In summary, the selection of surgical approach for locally advanced thymoma must follow the principle of “prioritising oncological efficacy over minimally invasive benefits.” By comprehensively assessing tumour size, the degree of vascular invasion, and the specific anatomical structures invaded, patients can be precisely stratified into a group suitable for MIS, a “grey zone” group requiring cautious evaluation, and a high-risk group that must undergo open surgery. This patient selection strategy based on multidimensional evidence not only maximises the advantages of MIS but also fundamentally ensures the radical resection rate and long-term survival benefits for patients with locally advanced disease.

## 3 Precision of postoperative adjuvant radiotherapy: from “all stage III” to “high-risk stage III”

### 3.1 Evolution of evidence-based medicine

For a long time, postoperative radiotherapy (PORT) has been regarded as the cornerstone of comprehensive treatment for locally advanced thymoma, particularly for patients with Masaoka-Koga stage III and IVa; traditional views tend to favour using radiotherapy to reduce the risk of local recurrence and compensate for potential inadequacies in surgical resection (37–39). However, with the refinement of minimally invasive surgical techniques and the deepening understanding of the biological behaviour of thymic tumours, this indiscriminate traditional approach is increasingly being questioned. Due to the lack of high-level evidence from prospective randomised controlled trials (RCTs), significant controversy has persisted regarding the survival benefit of PORT in completely resected (R0) stage II and III thymomas (38–40). In recent years, research data based on large-scale population databases and multicentre cohorts have presented a clear trend of narrowing indications supported by emerging evidence, prompting a shift in the focus of clinical decision-making from relying solely on anatomical staging for prophylactic irradiation to integrating recurrence risk stratification for precision treatment.

Accumulating clinical evidence supports narrowing the indications for PORT, particularly for patients with completely resected Masaoka-Koga stage II disease, where the survival benefit of PORT appears increasingly limited. The latest single-centre retrospective study indicates that for patients with radically resected stage II thymoma, whether in the IIa or IIb subgroup, PORT did not bring a significant benefit in DFS (41). This finding is strongly supported by a propensity score matching (PSM) study based on the SEER database; research by Shi et al. demonstrated that among patients with Masaoka-Koga stage IIB thymoma, PORT did not significantly improve OS or cancer-specific survival (CSS) (42). Furthermore, analysis of a large-scale database from the Japanese Association for Research on the Thymus (JART) also showed that PORT did not improve RFS or OS in patients with stage II and III thymoma, with benefits mainly limited to patients with thymic carcinoma (43). Retrospective analysis by the Chinese Alliance for Research in Thymomas (ChART) similarly pointed out that for R0-resected stage I to III thymomas, PORT did not improve OS or DFS and was even associated with a poorer prognosis in certain subgroup analyses; this may be related to selection bias in retrospective studies where “high-risk patients are more inclined to receive radiotherapy” (39). These findings collectively indicate that in the context of R0 resection, the necessity of routine radiotherapy based solely on staging (particularly stage II) is diminishing, and the treatment boundary is contracting towards high-aggressive subtypes.

However, controversy persists due to the significant heterogeneity of study designs and data sources; some high-quality evidence still supports the application of PORT in specific high-risk groups. For instance, an analysis of the International Thymic Malignancy Interest Group (ITMIG) database by Rimner et al. showed that PORT was independently associated with longer overall survival in patients with completely resected stage II and III thymoma (44). Similarly, a study by Jackson et al. based on the NCDB database also reported that PORT significantly improved the overall survival rate of patients with stage IIB and III thymoma, and this benefit persisted in patients with R0 resection (45). The latest meta-analysis also suggests that PORT can improve the OS of patients with completely resected Masaoka-Koga stage II/III thymoma, although it has no significant effect on DFS (46). Additionally, advanced radiotherapy technologies (such as 3D-CRT or IMRT) may bring survival benefits compared to conventional radiotherapy, suggesting that the evolution of radiotherapy techniques may also be an important variable affecting study conclusions (37). This contradictoriness in research results may stem from the inherent defects of retrospective data, such as the lack of detailed records regarding resection margin status, heterogeneity in WHO histological classification, and differences in radiotherapy doses (47, 48).

An in-depth analysis of the drivers behind this evidential shift reveals that the WHO histological classification and improvements in the quality of MIS have played key roles. With the deepening understanding of the biological behaviour of thymic epithelial tumours, WHO classification has been confirmed as an important prognostic factor independent of stage; the indolent characteristics of low-risk histological types (such as types A, AB, and B1) weaken the necessity for adjuvant radiotherapy (39, 49). Meanwhile, the maturation of MIS techniques has not sacrificed oncological efficacy; the studies summarised in Section 2.2 indicate that MIS can achieve R0 resection rates (100%) and long-term survival rates comparable to open surgery in locally advanced thymoma, which reduces the reliance on postoperative “salvage” radiotherapy caused by incomplete surgical resection. However, it must be critically pointed out that the vast majority of current evidence comes from retrospective studies, which harbour selection bias; that is, patients receiving PORT often possess adverse prognostic factors such as later stages, higher histological grades, or positive margins (38, 48, 50). Therefore, the traditional mode of deciding on PORT solely based on Masaoka-Koga staging has revealed limitations. Future treatment decisions should integrate resection margin status, histological subtypes, and molecular features more to integrate biological risk factors into the foundational stage-oriented framework (42, 47).

### 3.2 Identification of key risk stratification factors

In the clinical management of locally advanced thymoma, relying solely on anatomical staging is often insufficient to accurately predict recurrence patterns and guide adjuvant treatment decisions. Therefore, systematically identifying independent prognostic factors that determine the value of PORT is crucial for promoting the refinement of treatment models by implementing risk-oriented parameters into the traditional stage-oriented system. Although surgical resection remains the cornerstone of treatment, multiple studies based on large-scale population databases indicate that the survival benefits of PORT are not equally distributed among all patients but are significantly concentrated in subgroups with specific high-risk characteristics (51, 52). By integrating surgical resection status, intrinsic tumour biological behaviour, and local anatomical extent of invasion, clinicians can construct multidimensional risk stratification models to more precisely screen patient populations likely to derive the maximum survival benefit from adjuvant radiotherapy (52, 53).

#### 3.2.1 Surgery-related factors

The completeness of surgical resection (R status) intrinsically dictates the necessity of postoperative radiotherapy (PORT), serving as the foundational driver for adjuvant treatment decisions. Rather than applying PORT uniformly across all stages, clinical evidence strongly mandates stratifying patients based on residual disease. For patients with microscopic (R1) or macroscopic (R2) residual disease, PORT is unequivocally necessary. Comprehensive data from large-scale cohorts, including the ESTS and ChART databases, consistently demonstrate that PORT acts as a critical salvage therapy for incomplete resections. It significantly mitigates high recurrence risks and dramatically improves long-term outcomes, such as elevating 5-year overall survival rates from approximately 45-60% to 75-100% (52–54). Furthermore, multivariate analyses indicate that PORT effectively compensates for the local recurrence hazards introduced by minimal residual disease (55). Conversely, for patients who achieve complete (R0) resection, the survival benefit of PORT is highly nuanced and limited. While routine PORT in completely resected early-stage disease (e.g., Masaoka-Koga I/IIA) fails to provide significant survival advantages, it simultaneously exposes patients to unwarranted radiation-related toxicities (52, 56–58). Therefore, we conclude that PORT must be mandated as a standard component of care for all R1/R2 resections to optimise local control. For those achieving R0 resection, however, the decision to administer PORT cannot be justified solely by baseline anatomical staging; it must be judiciously individualised in conjunction with tumour biological characteristics to prevent detrimental overtreatment.

#### 3.2.2 Tumour biological factors

Beyond surgical margins, the intrinsic biological aggressiveness of the tumour—defined primarily by the WHO histological classification and tumour burden—serves as a critical determinant in identifying which patients will truly benefit from PORT. Evidence demonstrates that tumour biology frequently overrides broad anatomical staging in predicting local recurrence (53, 59, 60). Indolent histological subtypes (WHO types A, AB, and B1) demonstrate a remarkably low propensity for local relapse; consequently, applying PORT to these cohorts yields no survival advantage and merely subjects them to unnecessary radiation toxicity. In stark contrast, aggressive histological subtypes (WHO types B2, B3, and thymic carcinoma) exhibit inherently higher invasive potential (52). In these high-risk cohorts, PORT is instrumental in significantly prolonging long-term survival, demonstrating particularly pronounced benefits in patients with concurrent myasthenia gravis (MG) (60). Furthermore, quantitative tumour burden, typically reflected by tumour size, independently modulates recurrence risk. Tumours exceeding critical size thresholds (e.g., ≥ 55 mm or > 6 cm) correlate with an augmented risk of microscopic local invasion. Survival analyses confirm that PORT specifically confers overall survival advantages to patients harbouring these larger tumours, whereas those with smaller lesions derive no such systemic benefit (52, 58, 61). Consequently, we conclude that PORT decision-making must be fundamentally driven by biological aggressiveness. Adjuvant radiotherapy should be strongly recommended for tumours displaying high-risk WHO histologies (B2, B3, and C) and significant tumour burden (≥ 55 mm), while patients with indolent, small-volume tumours should be safely spared from adjuvant radiation.

#### 3.2.3 Extent of tumour invasion

The specific anatomical extent and nature of local tumour invasion provide a vital tertiary layer of risk stratification, critically informing both the indication for and the precise targeting of PORT. Broad staging classifications often fail to account for the heterogeneous prognostic impact of specific organ involvement (61, 62). Evidence demonstrates that the invasion of distinct anatomical structures dictates differing recurrence patterns and therapeutic sensitivities. For instance, direct pulmonary invasion is a profound risk factor associated with diminished disease-free and overall survival, necessitating intensified local control through PORT. Conversely, major vascular involvement, such as the invasion of the superior vena cava or innominate vein, may indicate a heightened need for systemic adjuvant therapies rather than isolated local irradiation (62). Furthermore, the breadth of invasion—quantified by the number of involved organs—proportionally increases the risk of extensive microscopic spread, thereby influencing adjuvant therapy choices (59). Crucially, understanding these specific invasion patterns directly shapes the definition of the radiotherapy target volume. Because thymic tumours predominantly fail via pleural dissemination or distant metastasis rather than regional lymphatic spread, comprehensive analyses reveal that locoregional recurrences are overwhelmingly out-of-field (63, 64). Therefore, we conclude that the evaluation of tumour invasion must focus on specific structural involvement rather than generic ‘T’ staging. To optimise the therapeutic ratio, PORT target volumes should be meticulously restricted to the tumour bed and immediately adjacent involved structures, actively avoiding broad, prophylactic nodal irradiation that increases toxicity without mitigating primary failure patterns.

### 3.3 Radiotherapy techniques and doses

With the indications for PORT now clearly based on multidimensional risk stratification, the selection of radiotherapy techniques and dose optimisation have become the core of balancing efficacy and toxicity. Although traditional two-dimensional or three-dimensional conformal radiotherapy (3D-CRT) can cover the tumour bed to a certain extent, the conformality of its dose distribution is relatively poor. This often results in the exposure of organs at risk (OARs) such as the heart, lungs, and oesophagus to unnecessary high-dose irradiation, thereby increasing the risks of radiation pneumonitis, pericarditis, and late cardiovascular events (65, 66). Given that patients with thymoma usually have a long life expectancy, the impact of late radiation toxicity—particularly the risks of cardiac toxicity and secondary malignant neoplasms (SMNs)—on quality of life cannot be ignored; therefore, implementing modern high-precision radiotherapy techniques to optimise the therapeutic ratio is essential (67, 68).

Regarding the application and comparison of modern radiotherapy techniques, intensity-modulated radiotherapy (IMRT) and volumetric modulated arc therapy (VMAT) have gradually replaced traditional techniques to become the mainstream. Multiple dosimetric studies have confirmed that compared with 3D-CRT, IMRT and VMAT can significantly improve the conformity index (CI) and homogeneity index (HI) of the target area while concurrently reducing the radiation dose to the lungs, heart, and breasts (69–71). For instance, a dosimetric analysis focussing on postoperative radiotherapy showed that compared with 3D-CRT, VMAT technology can significantly reduce the mean dose to the lungs (VMAT 10.8 Gy vs. 3D-CRT 13.5 Gy) and the heart (VMAT 8.6 Gy vs. 3D-CRT 12.4 Gy), thereby effectively lowering the risk of radiation pneumonitis and pericarditis (65). Furthermore, for female patients, the adoption of IMRT techniques with non-coplanar field settings (such as sagittal coplanar fields or fan-shaped fields) can significantly reduce the dose to the breasts, which is of great significance for reducing the risk of secondary breast cancer in young female patients (68). Similarly, while maintaining target coverage comparable to IMRT, VMAT technology can reduce monitor units (MU) and treatment time, thereby improving treatment efficiency (70).

In contrast, proton beam therapy (PBT), as an emerging technology, offers significant dosimetric advantages in protecting OARs by virtue of its unique physical property, the Bragg peak. Existing evidence supports that compared with IMRT, PBT can further reduce the mean doses to the heart, lungs, and oesophagus, and reduce the predicted risk of secondary malignant neoplasms (such as lung cancer, breast cancer, and esophageal cancer) by approximately half (67, 72, 73). Specifically, a prospective study indicated that compared with IMRT, double-scattered proton beam therapy (DS-PBT) reduced the mean heart dose by 36.5% (15.3 Gy vs. 22.8 Gy), the mean lung dose by 33.5%, and the mean esophageal dose by 60% (74). This dose reduction translates into clinical benefits; model predictions show that using PBT instead of photon radiotherapy can avoid the occurrence of approximately 5 excess secondary malignant neoplasms per 100 patients (67). Further research points out that PBT using pencil beam scanning (PBS) technology is superior to double-scattering techniques and photon techniques in reducing the risk of secondary malignant neoplasms, with particularly significant risk reductions for the breasts, lungs, and oesophagus (75). Additionally, in cases with complex anatomical structures or involving pericardial invasion, PBT can significantly reduce the dose to cardiac substructures (such as the left anterior descending coronary artery and left ventricle), thereby lowering the probability of major adverse cardiac events (MACE) and chronic heart failure (CHF) (71, 76, 77).

Regarding radiotherapy dose prescription, the current consensus emphasises individualised adjustment based on resection status and tumour staging. According to the DELPHI consensus of the French RYTHMIC network and the latest evidence-based medical data, the recommended total dose for postoperative adjuvant radiotherapy is 50 Gy for completely resected (R0) Masaoka-Koga stage IIb/III thymoma (78). If microscopic residual disease (R1) exists, it is suggested to increase the dose to 54 Gy; whereas for macroscopic residual disease (R2) or unresectable lesions, the dose needs to be further increased to 60 Gy or even higher (67, 77, 78). For unresectable locally advanced large-volume tumours (volume ≥ 200 cm³), the use of IMRT technique with a “Target-Ring” dose escalation strategy to increase the GTV dose to 70 Gy has shown a high objective response rate (84.6%) and favourable safety in a single-centre study (79). However, for early-stage completely resected cases, dose escalation has not shown significant survival benefits. A systematic review pointed out that in the context of postoperative adjuvant therapy, doses exceeding 50 Gy did not bring better survival rates, suggesting that for low-risk patients, the standard dose of 45–50 Gy is sufficient to control subclinical lesions (80).

Although high-dose radiotherapy may theoretically improve local control rates, the dose-response relationship remains controversial. Some studies indicate that in unresectable thymic epithelial tumours, patients receiving doses ≥ 54 Gy have significantly better PFS and OS than the low-dose group (<54 Gy), suggesting that 54 Gy may be the lower dose limit for definitive radiotherapy (81). However, studies have also explored the feasibility of dose de-escalation, finding that in completely resected stage II–III thymoma, reducing the adjuvant radiotherapy dose to 30–35 Gy (in 10 fractions) did not significantly lower the 3-year progression-free survival rate and resulted in lower toxicity, providing a new perspective for “lightweight” radiotherapy in low-risk patients (82). From this, it is speculated that for patients of different risk levels, the radiotherapy dose should be optimised towards low toxicity as much as possible under the premise of ensuring local control.

In summary, radiotherapy strategies for locally advanced thymoma are developing towards precision and individualization. Although modern photon technologies (IMRT/VMAT) have significantly improved dose distribution, proton therapy demonstrates greater potential in reducing the risk of late toxicity, making it particularly suitable for young patients or those at high cardiac risk (67, 71). Future research needs to further verify the long-term benefits of different dose fractionation modes and emerging radiotherapy technologies in specific subgroups through prospective clinical trials, so as to fill the gaps in current evidence regarding the optimal dose and technology selection (78).

## 4 Construction of an individualised treatment decision framework: integrating surgery and radiotherapy

### 4.1 Overview of the decision-making process

**Figure 1:**
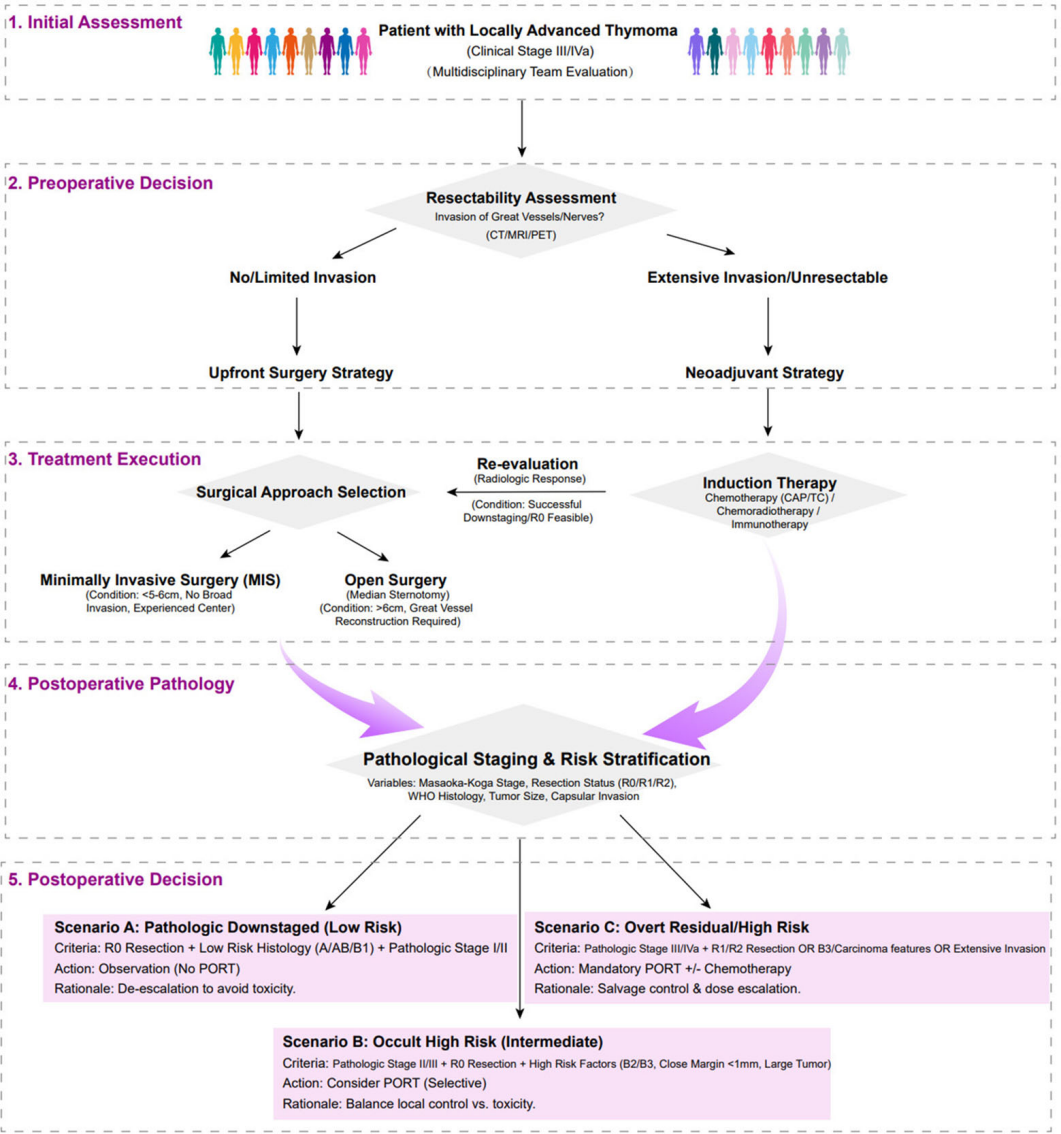
Integrated decision-making algorithm for locally advanced thymoma. The process is divided into two stages: preoperative resectability assessment guiding the choice between upfront surgery (MIS or Open) and neoadjuvant therapy; and postoperative risk stratification based on pathological features guiding adjuvant radiotherapy (PORT) decisions. MIS, Minimally Invasive Surgery; PORT, Postoperative Radiotherapy; R0/R1/R2, Resection status.

Treatment decision-making for locally advanced thymoma is a dynamic, continuous process spanning preoperative assessment, intraoperative adjustment, and postoperative management. Although the traditional Masaoka-Koga staging system has historically guided clinical practice, it relies primarily on postoperative pathological results, making it difficult to precisely guide the selection of MIS or the intervention of neoadjuvant therapy before the initiation of treatment (83, 84). Therefore, this review proposes a “Two-Stage Dynamic Precision Model,” aiming to organically integrate multimodal imaging features, the feasibility boundaries of minimally invasive surgical techniques, and postoperative pathological risk factors (**Figure 1**).

The first stage of decision-making (preoperative to intraoperative) focuses on the “dynamic assessment of resectability and downstaging conversion.” The core task of this stage is to rely on high-resolution CT and MRI imaging and, with the collaboration of a multidisciplinary team (MDT), precisely define the anatomical relationship between the tumour and great vessels (such as the superior vena cava and aorta) as well as the phrenic nerve. For cases evaluated as potentially resectable by imaging, the decision-making focus lies in assessing the feasibility and safety of MIS, striving to reduce surgical trauma without compromising oncological efficacy (R0 resection); whereas for cases where imaging suggests difficulty in achieving R0 resection, conversion strategies such as neoadjuvant chemotherapy or immunotherapy need to be initiated, with the aim of creating surgical opportunities through tumour downstaging.

The second stage of decision-making (postoperative) shifts to “adjuvant treatment stratification based on residual risk.” This stage optimises the traditional staging-based approach by integrating quantitative grading of recurrence risk based on postoperative pathological features (including WHO histological classification, resection margin status, and microvascular invasion). By identifying “pathologically downstaged” low-risk patients to exempt them from unnecessary radiotherapy, while precisely identifying occult high-risk populations accompanied by high-risk histological features or minimal residual disease for intensified treatment, this approach aims to reduce treatment-related long-term toxicity while maximizing the reduction of recurrence risk.

### 4.2 Initial strategy selection based on preoperative assessment

#### 4.2.1 Assessment of resectability and selection of surgical approach

Imaging evaluation is the primary step in formulating surgical strategies. Contrast-enhanced computed tomography (CT) remains the standard modality for evaluating the relationship between anterior mediastinal masses and surrounding structures, capable of displaying tumour size, location, and the relationship with adjacent mediastinal structures (85). For cases where CT display is unclear or cystic lesions are suspected, magnetic resonance imaging (MRI) possesses irreplaceable advantages in distinguishing solid tumours from cysts and evaluating microvascular invasion, particularly in its ability to more accurately depict the integrity of the tumour capsule (85, 86). At the same time, 18F-fluorodeoxyglucose positron emission tomography (18F-FDG PET/CT) not only aids in distinguishing thymoma from lymphoma (lymphoma usually exhibits higher SUV values), but its metabolic parameters can also assist in predicting the histological grade and invasiveness of the tumour (86, 87). For instance, a study by Zhou et al. indicated that a diagnostic model constructed by integrating parameters such as SUVmax and maximum tumour diameter can effectively enhance the differential diagnosis capability for invasive thymic epithelial tumours (87).

When evaluating the feasibility of MIS, “surgical ‘no-go’ zones” and “grey zones” must be strictly defined based on imaging features. According to the expert consensus of the Society of Thoracic Surgeons (STS), surgical resection should include en bloc resection of the tumour, residual thymus, and perithymic fat (86). If imaging shows extensive invasion of bilateral phrenic nerves, it is usually considered a contraindication for surgery, as bilateral phrenic nerve resection would lead to severe respiratory complications (86). However, for unilateral phrenic nerve involvement, resection can be performed provided the patient’s pulmonary function permits (86). Regarding great vessel invasion, the STS consensus points out that invasion involving structures such as the superior vena cava or innominate vein is not an absolute contraindication for MIS; however, in many cases, open surgery (such as median sternotomy) is still regarded as the standard approach to ensure the safety of vascular reconstruction and complete resection (R0) (86). Regarding tumour size, although there is no absolute threshold, tumour size (e.g., >5 cm) is included in considerations within TNM staging recommendations (86); nevertheless, the consensus by Liou et al. points out that as long as R0 resection can be safely achieved, MIS is applicable to stage I–II and even carefully selected stage III cases (86).

The indications for preoperative biopsy remain strictly defined. Although a large-sample analysis based on the NCDB database by Powell et al. pointed out that preoperative biopsy did not significantly reduce the OS of patients with early-stage thymoma, challenging the traditional concern that biopsy leads to tumour dissemination (88); the STS expert consensus still recommends that for thymic tumours with typical imaging features and assessed as resectable, unnecessary biopsy should be avoided to prevent potential seeding risks. Biopsy should be limited to cases where direct resection is not feasible, differentiation from lymphoma is difficult, or neoadjuvant therapy is planned (86). In summary, we recommend establishing the following stratified decision-making process: first, use contrast-enhanced CT or MRI to assess the degree of invasion of great vessels and phrenic nerves; second, combine with PET-CT to exclude distant metastasis and lymphoma; finally, the multidisciplinary team (MDT) should prudently recommend MIS or open surgery based on the surgeon’s experience curve, on the premise of ensuring R0 resection (86).

#### 4.2.2 The role of neoadjuvant therapy

For locally advanced tumours (Masaoka-Koga stage III/IVa) initially assessed as difficult to achieve R0 resection, neoadjuvant therapy aims to achieve “downstaging” and “conversion” (86, 89). Traditional platinum-based chemotherapy regimens (such as CAP, ADOC, or TC regimens) have been confirmed to effectively reduce tumour volume and improve R0 resection rates (90). For example, a study by Ma et al. compared different first-line chemotherapy regimens and found that the CAP regimen (cisplatin + doxorubicin + cyclophosphamide) could achieve an objective response rate (ORR) of more than 50% in thymoma (91).

In recent years, the paradigm of neoadjuvant therapy has been undergoing transformation. In terms of radiotherapy, Na et al. found through propensity score-matched analysis that compared with neoadjuvant chemotherapy (NCT) alone, neoadjuvant chemoradiotherapy (NCRT) significantly increased the R0 resection rate (93.3% vs 73.3%) and improved the tumour regression grade (TRG), demonstrating superior local control capabilities (92). Similarly, a phase II clinical trial by Xu et al. also supported the value of NCRT in improving the resection rate of locally advanced high-grade thymic tumours, reporting an R0 resection rate of 82.6% (93). In the field of immunotherapy, a phase II clinical trial conducted by Bang et al. showed that a neoadjuvant regimen of pembrolizumab combined with chemotherapy achieved a major pathologic response (MPR) rate of 46.4% and an R0 resection rate of 50% in locally advanced thymic epithelial tumours (94). However, this study also cautioned against the risks of immunotherapy in patients with thymoma: two patients with thymoma died due to the occurrence of fatal myocarditis, whereas patients with thymic carcinoma showed relatively better tolerability (94).

Furthermore, precision therapy targeting specific subtypes is also being explored. Research by Yu et al. found that for locally advanced type B thymomas (particularly types B1/B2), the preoperative use of glucocorticoids (prednisone) could significantly induce tumour regression (ORR reaching 64.8%), increase the success rate of MIS, and shorten operative time, with no severe surgical complications observed (95). Therefore, the selection of neoadjuvant therapy should be highly individualised: NCRT may be considered for cases pursuing maximal local control; for patients with thymic carcinoma, immunotherapy combined with chemotherapy may offer new opportunities; and for specific type B thymomas, glucocorticoids represent a low-toxicity, highly effective alternative (92, 94, 95).

### 4.3 Formulation of adjuvant treatment strategies based on postoperative pathological-surgical composite features

Although preoperative imaging assessment defines the initial treatment strategy for “locally advanced” disease, the precise postoperative pathological staging, histological subtype, and quality of surgical resection (R status) are the final determinants of adjuvant treatment intensity. For patients with locally advanced disease who have undergone MIS or neoadjuvant therapy, the decision regarding PORT should no longer simply follow traditional anatomical staging but should instead shift towards a dynamic adjustment model based on “Residual Risk.” Based on the composite features of postoperative pathology and the surgical procedure, we categorise patients into three scenarios for precise decision-making.

#### 4.3.1 Scenario A: pathologically downstaged—”unexpected” low risk

This scenario is defined as: preoperative imaging assessment suggested locally advanced disease (e.g., suspicious invasion of the lung or pericardium), but following complete resection (R0) via MIS or open surgery, pathology confirmed only capsular invasion (Masaoka stage II/T1b) or limited adhesion to adjacent organs (non-invasive), and the histology was a low-risk subtype (e.g., WHO type A, AB, or B1). For such “radiographically overstaged” patients, even if categorised as locally advanced preoperatively, a “de-escalation” strategy should be decisively implemented postoperatively, omitting PORT.

This strategy is supported by multiple large-scale studies. The systematic review and clinical guidelines by Falkson et al. point out that for completely resected early-stage thymoma (TNM stage I/Masaoka stage I-IIA), postoperative radiotherapy did not confer a survival benefit and instead increased the long-term risks of cardiac toxicity and secondary malignancies (96). A recurrence prediction model established by Liu et al. based on the ChART database further confirmed that for patients in the low-risk group (e.g., types A-B1), even with larger tumour volumes, as long as R0 resection is achieved, the 10-year recurrence-free survival rate is as high as 96.8%, indicating an extremely low risk of postoperative recurrence (97). Furthermore, research by Zhou et al. indicates that imaging modalities such as PET/CT may yield false positives regarding invasiveness, leading to preoperative overstaging (87); therefore, once postoperative pathology confirms a low-risk entity, there is no need for defensive overtreatment based on the preoperative “advanced” impression, and replacing adjuvant therapy with regular imaging follow-up is recommended.

#### 4.3.2 Scenario B: occult high risk—selective intensification strategy

This scenario covers the “grey zone” that is most controversial in clinical decision-making. It is defined as: preoperative assessment suggested locally advanced disease, R0 resection was performed, but postoperative pathology indicated the presence of high-risk histology (WHO type B2/B3), or the existence of a risk of a microscopically close margin (Close Margin, <1mm), particularly adjacent to preserved vessels or nerves. For this population, the decision regarding PORT requires weighing the pros and cons, and “margin-directed precision PORT” is recommended.

On one hand, high-risk histology itself is an independent predictor of recurrence. Research by Liu et al. categorised types B2 and B3 into the high-risk group, pointing out that their postoperative recurrence rate was significantly higher than that of the low-risk group (20.1% vs 2.7%), with recurrences mostly concentrated in the first 3 years postoperatively, suggesting that such patients require more aggressive intervention even after R0 resection (97). A meta-analysis by Tateishi et al. also supports that PORT can significantly improve OS in completely resected stage II/III thymomas, particularly in cases with high-risk recurrence factors (46). On the other hand, the STS surgical consensus by Liou et al. emphasises that “extremely close margins” left during MIS to preserve the phrenic nerve or blood vessels may harbour a risk of microscopic residual disease; although overall survival is similar between MIS and open surgery, for high-risk subtypes (B2/B3) or those with suspicious margins, PORT is an effective means to reduce local recurrence (86). Therefore, for Scenario B, the use of IMRT or proton therapy for local boost irradiation is recommended to balance local control with the protection of surrounding organs at risk (e.g., heart, lungs) (86).

#### 4.3.3 Scenario C: overt high risk—salvage treatment strategy

This scenario involves refractory locally advanced thymomas. It is defined as: R0 resected advanced high-risk thymomas (Masaoka stage III/IVa, WHO type B3), or any form of incomplete resection (R1/R2). For such patients, surgery alone cannot meet the need for disease control, and PORT should be regarded as a “mandatory standard component”.

For patients with thymoma who underwent R1 (microscopic residual) or R2 (macroscopic residual) resection, regardless of histological type, the latest STS expert consensus and the guidelines by Falkson et al. strongly recommend postoperative radiotherapy to control local disease (86, 96). Research by Fan et al. also confirmed that for patients with locally advanced Masaoka stage III thymoma that could not be completely resected, the administration of definitive radiotherapy (dRT, ≥54 Gy) significantly improved the 5-year overall survival rate (65.7% vs. 26.8%) and locoregional recurrence-free survival rate (98). A study by Leuzzi et al. based on the ESTS database further pointed out that for locally advanced thymomas at stage pT3, adjuvant therapy (including radiotherapy) was significantly associated with better cancer-specific survival (CSS) (99). For cases with R2 resection or unresectable disease, the radiotherapy dose typically needs to be increased to 60–70 Gy to control macroscopic residual disease (86, 96). Furthermore, for high-risk cases of stage IVa disease accompanied by pleural dissemination, in addition to radiotherapy, intrapleural local therapy (such as hyperthermic perfusion chemotherapy) or systemic therapy may be considered as part of comprehensive management (96).

#### 4.3.4 Summary: dynamic and multimodal decision model

In summary, the decision for adjuvant treatment in locally advanced thymoma should not be a formulaic routine, but rather a dynamic calibration process based on “risk orientation”. From the preliminary staging via preoperative imaging to the precise management of margins during MIS, and finally to the definitive determination of biological behaviour (WHO classification) and resection quality (R status) by postoperative pathology, treatment strategies must be adjusted alongside the modification of risk levels. Through this refined stratification—decisively implementing de-escalation for patients with pathologic downstaging (Scenario A), delivering precision radiotherapy for those with occult high risk (Scenario B), and administering high-dose salvage radiotherapy for those with macroscopic residual disease or advanced high-risk thymoma (Scenario C)—we can maximally avoid overtreatment in low-risk patients while ensuring high-risk patients receive sufficient treatment intensity, thereby optimizing individualised patient care.

## 5 Challenges, controversies, and future directions

Although MIS and precision radiotherapy have advanced the management of locally advanced thymoma, clinical decision-making remains limited by a lack of high-level evidence. First, existing decisions regarding PORT mainly rely on analyses of retrospective databases (such as SEER, ChART, and ITMIG); these studies inevitably contain selection bias, and conclusions regarding whether patients with completely resected (R0) Masaoka-Koga stage IIB and III disease can derive definite survival benefits from PORT remain inconsistent. Some large-sample studies from 2024 even point out that PORT did not improve DFS or OS in patients with specific stages (42, 52). Second, at the surgical level, although techniques such as RATS have expanded to locally advanced cases, for complex cases with larger tumour diameters (>5 cm) or accompanied by vascular invasion, how to balance the advantages of MIS with tumour capsule integrity and the risk of pleural dissemination remains a focal point of debate in the surgical field; a few studies suggest that the MIS approach may be associated with poorer long-term DFS (33, 76). Furthermore, due to the indolent biological behaviour of thymoma, the follow-up duration in most existing studies is insufficient to capture late recurrence events, which also limits the accurate evaluation of the long-term oncological efficacy of different treatment modes (100).

The future treatment paradigm for locally advanced thymoma will accelerate its evolution by implementing biological risk-oriented parameters into the foundational anatomical stage-oriented model. In the field of radiotherapy, multicentre prospective studies are urgently needed to validate the long-term advantages of advanced technologies such as PBT in reducing the risks of cardiac toxicity and secondary malignancies, and to further explore dose de-escalation strategies for radiotherapy to optimise the therapeutic ratio (82). Regarding systemic therapy, with breakthroughs in molecular biology, integrating genomic characteristics (such as *GTF2I* and *TP53* mutations) with immune biomarkers will help guide the perioperative application of immune checkpoint inhibitors (ICIs) or targeted drugs; recent studies have also revealed the unique value of glucocorticoids as neoadjuvant therapy in specific type B thymomas (95, 101–103). Ultimately, future directions should aim to construct dynamic individualised prediction models by integrating radiomics, pathological subtypes, and surgical parameters through MDT, thereby maximally preserving patients’ organ function and quality of life while ensuring radical tumour eradication.

## 6 Conclusion

The management of locally advanced thymoma is transitioning towards individualised precision treatment by implementing biological risk-oriented parameters into the foundational anatomical staging system. By systematically reviewing recent evidence-based medical data, this review clarified the oncological safety of MIS in selected locally advanced cases, confirming that it can serve as an effective alternative to open surgery in experienced centres, laying a solid foundation for reducing treatment trauma and accelerating recovery. Simultaneously, addressing the controversy surrounding PORT, this paper established a multidimensional risk stratification logic based on resection status, histological subtype, and tumour burden, proposing that the application of PORT should not be a “one-size-fits-all” approach but should be strictly based on detailed postoperative pathological risk assessment to strike a balance between improving local control and reducing late radiation toxicity. This review clarifies the boundaries of indications for minimally invasive techniques and radiotherapy, and emphasises that optimal outcomes depend on the comprehensive consideration of surgical feasibility, pathological features, functional imaging, and patient preferences by the MDT. Finally, with the further integration of molecular biomarkers and advanced radiotherapy technologies, the diagnosis and treatment paradigm for locally advanced thymoma will continue to evolve, ultimately improving oncological outcomes and patient quality of life.
